# Probing rare von Willebrand disease–causing mutations in the D4 and C-domains of von Willebrand factor

**DOI:** 10.1016/j.rpth.2025.102922

**Published:** 2025-06-06

**Authors:** Golzar Mobayen, Sammy El-Mansi, Alain Chion, Thomas D. Nightingale, Thomas A.J. McKinnon

**Affiliations:** 1 Department of Immunology and Inflammation, Centre for Haematology, Imperial College Academic Health Science Centre, Hammersmith Hospital, London, UK; 2 Department of Medicine, Centre for Microvascular Research, William Harvey Research Institute, Queen Mary University of London, London, UK; 3 Irish Centre for Vascular Biology, School of Pharmacy and Biomolecular Sciences, Royal College of Surgeons in Ireland, Dublin, Ireland

**Keywords:** von Willebrand factor, von Willebrand disease, variant analysis, bleeding, mutations

## Abstract

**Background:**

von Willebrand disease (VWD) is characterized by absence or reduction of plasma von Willebrand factor (VWF) levels or reduced protein function. While the spectrum of causative VWD mutations is vast, there has been limited characterization of variants occurring within the D4-C6 domains of VWF that comprise the C-terminal portion of the molecule.

**Objectives:**

In this study, we investigated the impact of 9 putative low-frequency VWD-causing variants on VWF function.

**Methods:**

Variants were generated by site-directed mutagenesis and expressed in human embryonic kidney (HEK)293(T) cells and analyzed for expression, intracellular storage, and multimeric profile. The ability of Arg2379Cys to form dimers was assessed using an A2-CK fragment of VWF.

**Results:**

Arg2379Cys, Ser2497Pro, and Cys2639Tyr had significantly reduced secretion from HEK293T cells, while the other mutations all failed to be secreted. While cotransfection with wild-type VWF appeared to rescue expression, cotransfection with a deletion A1 construct demonstrated that only the Gly2044Asp, Glu2343Val, Ser2497Pro, and Cys2693Tyr variants could be rescued. All the variants failed to form appreciable pseudo–Weibel-Palade bodies in HEK293 cells and showed abnormal multimers in cell lysates. The Arg2379Cys variant could be overexpressed by only formed monomers and some dimers. Analysis with a VWF-A2CK protein demonstrated that in the homozygous state Arg2379Cys behaves likes a type 2A variant, while it is likely to be a type 1 variant in the heterozygous state.

**Conclusion:**

These data show that variants within the C-terminal region of VWF can dramatically impact proper VWF expression and can different impacts on VWF depending on homozygosity or heterozygosity.

## Essentials

• VWD causing variants in the D4 and C-domains are rarely studied.
• A small panel of VWD variants were investigated for expression and function.
• The variants affected VWF expression, multimer formation and storage.
• Arg2379Cys is a novel type 2A variant in the homozygous state, but type 1 if heterozygous.

## 1 Background

von Willebrand factor (VWF) is a large plasma glycoprotein (GP) essential to normal hemostasis by acting as the carrier molecule for coagulation factor (F)VIII and supporting platelet adhesion and aggregation at sites of vascular injury [1,2]. VWF is synthesized exclusively in endothelial cells and megakaryocytes where it is stored in Weibel–Palade bodies (WPBs) and α-granules, respectively [3,4]. Critical to its function is its multimeric size, with the largest multimers being the most active [5]. VWF monomers comprise a series of repeating domains arranged in the order: D1D2-D′D3-A1A2A3-D4C1C2C3C4C5C6CK, with many of the domains having functional characterized roles [6]. The D1D2 domains comprise the propeptide and are essential for multimer formation [7]; the cysteine knot (CK) domains contain cysteine residues required for dimer formation [8]; and the D′D3 domain contains the FVIII-binding sites and cysteine residues required for multimer formation [[9], [10], [11]]. The A1 and A3 domains bind to exposed collagen, and the A1 domain interacts with platelet GPIbα [[12], [13], [14], [15]]. The A2 domain harbors the A disintegrin and metalloprotease with thrombospondin motif repeats (ADAMTS)-13 cleavage site, with a docking site for ADAMTS-13 reported in the D4 domain [16,17]. Finally, the C4 domain presents an Arg-Gly-Asp sequence required for binding to platelet GPIIb/IIIa [18].

The clinical importance of VWF is highlighted by von Willebrand disease (VWD), which is generally caused by mutations in the *VWF* gene and is characterized by a bleeding phenotype, which can vary in severity from asymptomatic to life-threatening bleeding [19,20]. VWD is the result of either a quantitative or qualitative deficiency of VWF and can be broken down into 3 subtypes. Type 3 VWD is characterized by a total absence of VWF in the circulation, whereas type 1 represents reduced plasma levels. It is noteworthy that a small portion of patients with type 1 VWD do not have a mutation in the *VWF* gene [21]. Type 2 VWD is broken down into further subtypes and results from functional defects such as aberrant multimer formation (type 2A, group I), enhanced susceptibility to ADAMTS-13 cleavage (type 2A, group II), reduced collagen or platelet binding (type 2M), enhanced binding to GPIbα leading to increased proteolysis and clearance (type 2B), and reduced FVIII binding (type 2N) [19].

The spectrum of missense VWD-causing variants is vast, with mutations reported in every domain. While many of these are rare variants and often only appear in the heterozygous state, knowing where the mutation occurs can be useful for diagnosis and clinical management; however, genetic testing is not performed for the majority of cases [22]. Studies reporting on the phenotypic effects of variants on VWF expression and function using recombinant expression and *in vivo* systems provide significant information about the impact of the variant. Regardless of the frequency of a particular variant, such studies enhance our understanding of VWF biology and can in some cases help to guide the appropriate treatment [[23], [24], [25], [26], [27], [28], [29], [30], [31]]. However, many reports tend to focus on VWD causative variants in the N-terminal portion of the molecule, taken as the D1 to A3 domains, with much less attention paid to the C-terminal region; D4-CK and indeed our overall understanding of how this part of VWF functions is underexplored compared with the N-terminal domains. However, recently Dubois et al. [32] described 2 frame shift variants located in the D4 domain.

To address this, in this study, we selected rare 9 putative missense variants in the D4 and C1 to C6 domains, which are associated with VWD but have yet to been extensively characterized using *in vitro* studies. Using a recombinant expression system, we aimed to demonstrate that variants in the D4 and C-domains of VWF can significantly affect VWF expression and function and the impact of the causative mutation can be different depending on its heterozygous or homozygous status.

## 2 Methods

### 2.1 Variant selection

The gnomAD v4.1.0 database [33] was accessed via https://gnomad.broadinstitute.org/ and a search performed for VWF to show only “missense/inframe indel” variants, and data were saved as a CSV file. Variants falling outside the D4 to C6 domains (taken as residues 1873-2722) were discarded along with inframe and indel variants, leaving only missense mutations. Variants were selected at random and crossreferenced to the European Association for Haemophilia and Allied Disorders coagulation factor variant database to determine whether any clinical data were available and if any *in vitro* functional work had been performed. Additionally, basic literature searches were carried out using Pubmed and Google, searching for the mutant location with either single or triple letter amino acid code and either “VWF” or “VWD.” Variants that had previous *in vitro* characterization were excluded.

### 2.2 Expression of VWF mutants

Full-length VWF mutants were generated as previously described. Mutagenesis primers were designed using Primer X (https://www.bioinformatics.org/primerx/), and the QuikChange XL site-directed mutagenesis kit (Stratagene) was used to generate mutations in the vector pcDNA3.1-VWF-A2CK, which encodes for the VWF A2 through to CK domain. Following confirmation of the desired mutation, the A2-CK fragments was subcloned into our full-length VWF expression vector pcDNA3.1-FL-VWF using the restriction enzymes KpnI and AgeI. All mutations were reconfirmed by DNA sequencing. Wild-type (wt) and mutated VWF were then expressed in human embryonic kidney (HEK)293T cells as previously reported using 10 mM polyethylenimine as a transfection reagent. Three days posttransfection, the conditioned medium was collected, and cells were lysed in chilled lysis buffer (150 mM NaCl, 50 mM Tris, 5 mM EDTA, 1% Triton, 100×, pH7.4) for 5 minutes on ice. Lysates were then collected and centrifuged at maximum speed (13,500 rpm) for 10 minutes to remove cellular debris. For large-scale protein production, the conditioned media was concentrated approximately 20-fold using 100-kDa Amicon Ultra 15-mL centrifugation filter units (Millipore). For cotransfection experiments, HEK293T cells were transfected with the mutated VWF sequences alongside either pcDNA3.1-FL-VWF or the vector pcDNA3.1-FL-VWF-ΔA1 construct that lacks the A1 domain in a 1:1 ratio [34,35].

### 2.3 Immunofluorescence analysis of intracellular VWF

Immunofluorescence analysis of intracellular VWF was performed using HEK293 cells since HEK293T cells fail to form WPB-like granules. HEK293 cells expressing wt and mutant VWF constructs were fixed with 4% paraformaldehyde for 15 minutes in the dark. The fixed cells were washed 3 times with phosphate-buffered saline (PBS) and then permeabilized by the addition of 1% Triton (Sigma) diluted in PBS for 10 minutes at room temperature. Cell were subsequently washed and stained for VWF and the endoplasmic reticulum (ER) using sheep polyclonal anti-VWF antibody (fluorescein isothiocyanate; CiteAb) and mouse anti–protein disulphide isomerase antibody (F-4; Santa Cruz Biotechnology), both diluted to 1 μg/mL, for 1 hour at room temperature. Following staining, cells were washed 4 times with PBS and then stained with goat antimouse AlexaFluor-555 secondary antibody diluted 1 in 2000 for 20 minutes. After washing, coverslips were mounted on glass slides using mounting medium with 4',6-diamidino-2-phenylindole (Abcam). Images were acquired using the Zeiss LSM800 confocal microscope. Images are presented as a maximum intensity projection of 0.5 μm interval Z stacks.

### 2.4 Protein analysis

VWF levels in the media and lysate were quantified using an in-house VWF ELISA, and multimer distribution of VWF in the media and lysate was assessed using nonreducing 1.2% agarose gel electrophoresis, both as previously described [36,37]. Analysis of the VWF A2-CK protein was performed using sodium dodecyl sulfate (SDS) polyacrylamide gel electrophoresis (PAGE). Samples were diluted with NuPAGE lithium dodecyl sulfate Sample Buffer (4×; Invitrogen) supplemented with 7% β-mercaptoethanol (Sigma) as a reducing agent when required. Precast 4% to 12% Bis-Tris Gels (Invitrogen, ThermoFisher Scientific) were set up in Mini Gel Tanks (Invitrogen, ThermoFisher Scientific) with 1× NuPAGE 2-(N-morpholino)ethanesulfonic acid SDS Running buffer (Invitrogen, ThermoFisher Scientific). Samples were denatured for 10 minutes at 90 °C and then loaded onto the gel alongside a prestained 10- to 250-kDa marker (Cell Signaling). Electrophoresis was carried at 200 V for approximately 40 minutes. Following SDS-PAGE, proteins were transferred onto nitrocellulose membranes using the Bio-Rad Trans-Blot Turbo Transfer System, as per manufacturer protocol using the high molecular weight program for 15 minutes. Membranes were then blocked in PBS supplemented with 0.1% Tween-20 containing 2% skimmed milk (Sigma) on a roller for 1 hour at room temperature. Following blocking, membranes were incubated with polyclonal anti-VWF horseradish peroxidase (Dako) conjugated antibodies to detect VWF-A2-CK. Protein was detected using SuperSignal West Pico Plus Chemiluminescence substrate (ThermoFisher Scientific), and membranes were visualized using the iBright 1500 Imaging system (ThermoFisher Scientific).

### 2.5 Perfusion assays

Analysis of VWF function under flow was carried out essentially as previously described [24]. In brief, Ibidi VI^0.1^ slides were coated with 100 μg/mL human type III collagen (Southern Biosciences) overnight in a humidified chamber. Channels were washed 3 times with PBS before the addition of 2% bovine serum albumin/PBS for a minimum of 15 minutes. Plasma-free blood was prepared as previously described, with platelets rendered fluorescent by the addition of 10 mM DiOC_6_ (3,3'-dihexyloxacarbocyanine iodide; ThermoFisher Scientific). Plasma-free blood was supplemented with 2.5 μg/mL of either wt or mutant VWF and perfused over the collagen surface at 140 μL/min to give a final wall shear rate of 1500/s. Images of bound platelets were captured in real-time under an Olympus-CKX41–inverted fluorescent microscope linked to a Rollera XR camera. Platelet capture was analyzed using ImageJ software.

### 2.6 Structural models

Since there is no experimental structure for the VWF D4-C6 region, we used the Alphafold model for porcine VWF (AF_AFQ28833F1; https://www.rcsb.org/structure/AF_AFQ28833F1) as a template for modeling with Modeller software [38]. The porcine VWF Alphafold model is better than the human VWF Alphafold model in A3-CK region. A VWF-A3-C3 model (residues 1672-2496) and a VWF-C1-C6 model (residues 2256-2720) were generated and analyzed using Pymol software.

### 2.7 Data analysis

Data analysis was performed using Prism Software for Science software package (version 10.0; GraphPad Software). Results are expressed as SD with mean. The statistical significance of differences between groups was assessed using 1-way analysis of variance (anova) with multiple comparisons.

## 3 Results

### 3.1 The spectrum of D4 and C-domains missense variants

The D4 and C-domains of VWF comprise residues 1873 to 2722 and are encoded by exons 35 to 50 of the *VWF* gene. We initially interrogated the gnomAD database and found 1041 reported missense variants between amino acids 1873 and 2722 inclusive, representing mutation of 623 of 850 (73.1%) amino acids comprising the D4 domain through to the C6 domain. The distribution of variants did not differ significantly between domains, with each domain having between 70% and 79% of residues mutated (Table 1). Ultimately, for the purpose of this study, 9 single-point mutations that had been previously reported but uncharacterized in detail were chosen for further investigation: 4 were located in the D4 domain—Cys1950Tyr, Gly2044Asp, Cys2174Gly, and Glu2233Gln; 3 in the C2 domain—Cys2340Arg, Gly2343Val, and Arg2379Cys; and one each in the C4 and C6 domains—Ser2497Pro and Cys2693Tyr, respectively (Table 2) [[39], [40], [41], [42], [43]]. Two of the variants (Gly2044Asp and Cys2174Gly) were categorized as type 3 VWD with the remaining variants predicted to be type 1. Both PolyPhen-2 and SIFT scores predicted all mutations, with the exception of Ser2479Pro to be damaging or affect protein function (Table 2).

**Table 1:** Distribution of von Willebrand factor mutations in the D4 assembly and C-domains.

Domain	No. of residues	No. of mutations	Percentage of amino acids mutated
D4 assembly	382	276	72.3
C1	79	56	70.1
C2	69	50	72.5
Linker	26	20	76.9
C3	68	48	70.6
C4	81	64	79
C5	69	53	76.8
C6	76	56	73.7
	Total = 850	Total = 623	Average = 73.3

**Table 2:** Characteristics and *in silico* prediction of 9 von Willebrand disease causative mutations.

DNA change	Amino acid change	Domain	Clinical data				Genotype	Predicted VWD type	PolyPhen-2		SIFT		Reference
			VWF:Ag	VWF:RCo	VWF:CB	Multimers			Confidence score	Prediction	Score	Prediction	
c.5849G>A	Cys1950Tyr	D4 (VWD4)	23	14	—	—	Het	Type 1	1.00	PD	0.00	APF	[39]
c.6131G>A	Gly2044Asp	D4 (VWD4)	2	5	—	Absent	C/Het	Type 3	1.00	PD	0.00	APF	[40]
c.6520T>G	Cys2174Gly	D4 (C8-4)	1	-	—	—	Hom	Type 3	1.00	PD	0.00	APF	[41]
c.6697G>C	Glu2233Gln	D4 (TLI-4)	25	18	—	—	Het	Type 1	1.00	PD	0.00	APF∗	—
c.7018T>C	Cys2340Arg	C2	27	25	—	—	Het	Type 1	1.00	PD	0.00	APF	[42]
c.7028G>T	Gly2343Val	C2	45	41	—	—	Het	Type 1	1.00	PD	0.00	APF∗	[42]
c.7135C>T	Arg2379Cys	C2	29	35	—	—	Het	Type 1	0.00	PD	0.04	APF∗	[42]
c.7489TT>C	Ser2497Pro	C4	27	25	—	—	Het	Type 1	1.00	Benign	0.28	Tolerated	[42]
c.8078G>A	Cys2693Tyr	C6	46	64	42	Normal	Het	Type 1	1.00	PD	0.00	APF∗	[43]

APF, affect protein function; APF∗, affect protein function with low degree of certainty; C/Het, compound heterozygous; Het, heterozygous; Hom, homozygous; PD, probably damaging; RCo, Ristocetin cofactor; vWD, von Willebrand disease; vWF, von Willebrand factor.

### 3.2 Mutations in the D4 and C-domains affect VWF expression

**Figure 1:**
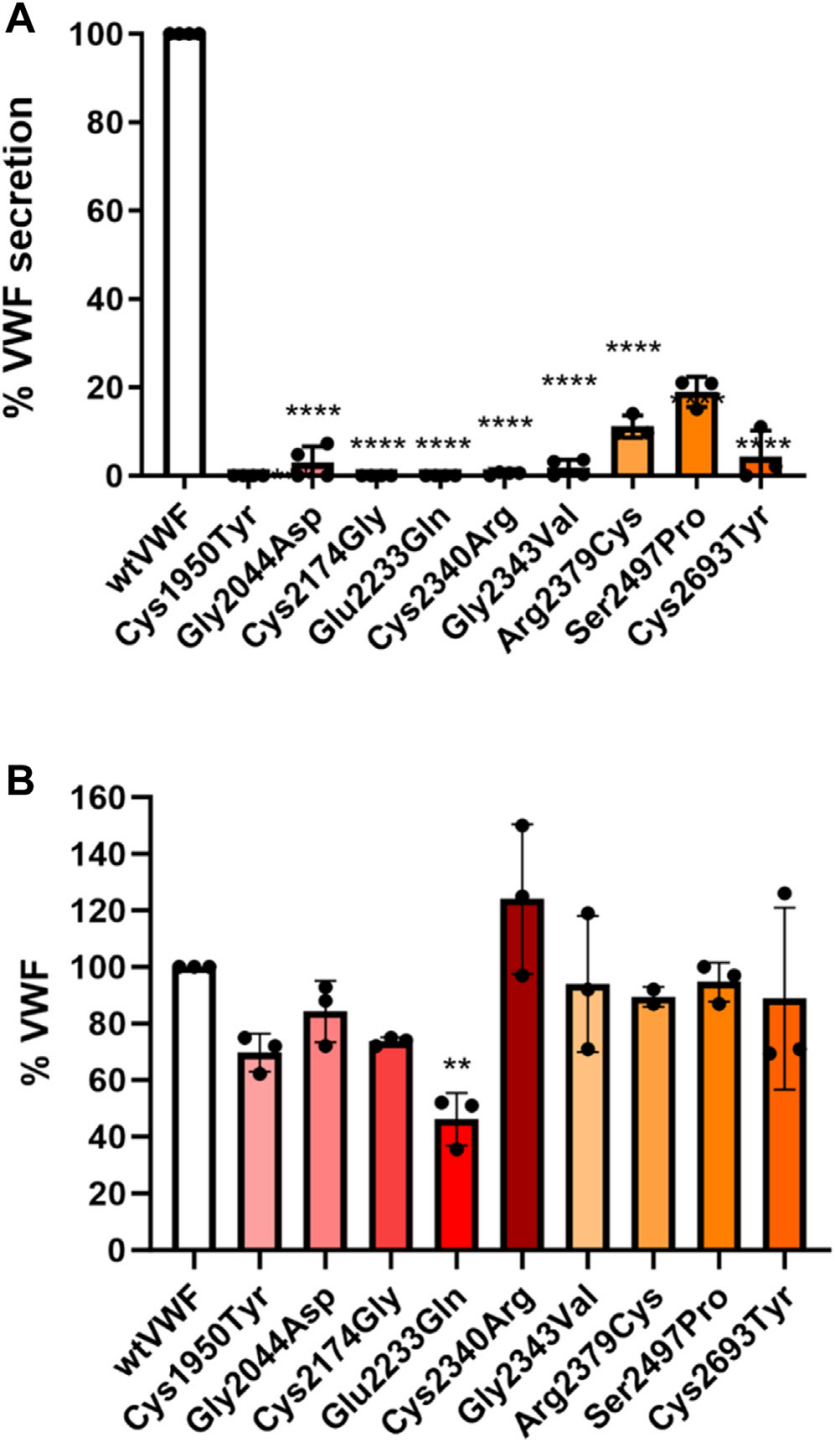
Homozygous expression of recombinant von Willebrand factor (VWF) mutants. HEK293T cells were transfected with expression vectors for the D4 and C-domain mutants. Conditioned media and lysates were harvested 72 hours posttransfection, and the concentration of VWF was quantified using ELISA. Results were calculated relative to wild-type (wt) VWF expression as a percentage (%). (A) VWF levels in the media was virtually abolished for all mutants except for Arg2379Cys, Ser2497Pro, and Cys2693Tyr. (B) VWF levels in cell lysates demonstrated comparable levels with wtVWF with the exception of Glu2233Gln, which demonstrated a significant decrease in VWF:Ag levels. (Error bars represent the mean SD of 3 biologic repeats performed in duplicate. ∗∗∗∗*P* ≤ .0001).

The selected mutations were generated in the expression vector pcDNA3.1-FL-VWF and transiently expressed in HEK293T cells and VWF levels in both media and lysate quantified by ELISA. Compared with wild-type (wt) VWF, the variants demonstrated significantly reduced secretion. In keeping with their type 3 VWD phenotype, virtually, no secretion was observed for the Gly2044Asp and Cys2174Gly variants (Figure 1A). No secretion was also observed for the Cys1950Y, Glu2233Gln, Cys2340Arg, Gly2343Val, and Cys2693Tyr variants, indicating a detrimental effect on expression (Figure 1A). Significantly reduced levels were observed with the Arg2379Cys and Ser2497Pro variants (10%-20% of normal). Interestingly, all mutant proteins were present in the cell lysate at a similar level to wtVWF, with the exception of VWF-Glu2233Gln (Figure 1B).

### 3.3 Increased expression of some of the VWD mutants cannot be rescued by coexpression of wtVWF

**Figure 2:**
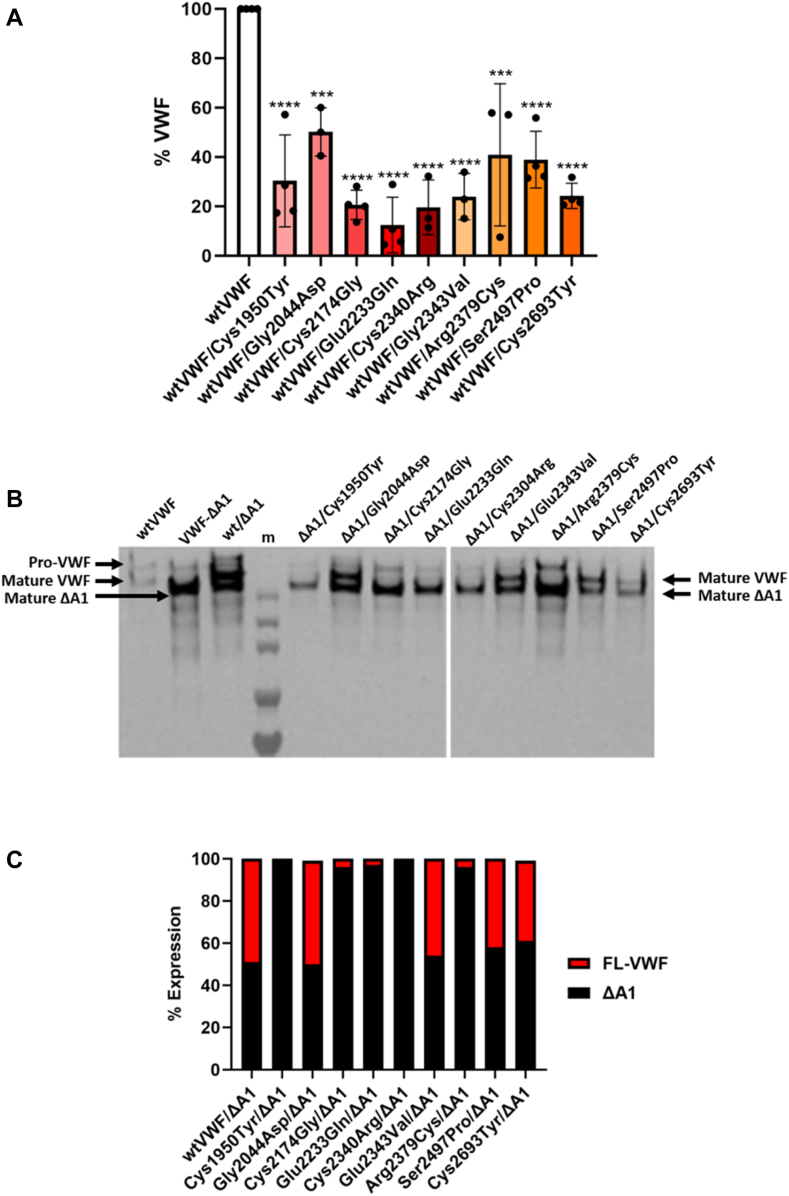
Heterozygous expression of recombinant von Willebrand factor (VWF) mutants. (A) HEK293T cells were transfected with expression vectors for wild-type von Willebrand factor (wtVWF) and the D4 or C-domain mutants. Conditioned media was harvested 72 hours posttransfection, and the concentration of VWF was quantified using ELISA. Results were calculated relative to wtVWF expression as a percentage (%). All mutants demonstrated rescued expression but were significantly reduced compared with wtVWF. (B) HEK293T cells were cotransfected with expression vectors for mutant VWF alongside VWF-ΔA1. At 72 hours posttransfection, conditioned media was collected and analyzed by SDS-PAGE and VWF was detected by western blot with anti-human VWF horseradish peroxidase–conjugated antibody. (C) The ratio between mutant and nonmutant monomer cotransfections were quantified using ImageJ. Mutants Gly2044Asp, Glu2343Val, Ser2497Pro, and Cys2693Tyr demonstrated rescued expression when cotransfected with VWF-ΔA1, while Cys1950Tyr and Cys2340Arg failed to express mutant VWF. Cys2174Gly, Glu2233Gln, and Cys2340Arg mainly expressed VWF-ΔA1 with minimal mutant protein expression. (Error bars represent the mean SD of 3 biologic repeats performed in duplicate. ∗∗∗*P* ≤ .001 ∗∗∗∗*P* ≤ .0001).

Since many of these mutations are likely to be heterozygous, transfections were repeated coexpressing wtVWF alongside the variant protein. Coexpression with wtVWF increased VWF secretion to varying extents, however, this rarely exceeded 50% of normal (Figure 2A). In order to investigate whether the secreted protein comprised a mixture of wt and variant protein, cotransfections were repeated where the full-length expression vector containing the desired mutation was transfected alongside a VWF-ΔA1 construct that lacks the A1 domain. As shown in Figure 2B, when run on reducing SDS-PAGE gel, the ΔA1 monomers migrate faster than full-length monomers, allowing a ratio of the 2 to be determined. When transfected alongside full-length VWF, the ratio of wtVWF:ΔA1 was approximately 1:1. The Gly2044Asp, Gal2343Val, Ser2497Pro, and Cys2693Tyr variants were secreted in an approximately 1:1 ratio, with VWF-ΔA1 showing that these could be incorporated into released VWF (Figure 2B, C). However, for the Cys1950Tyr, Cys2174Gly, Glu2233Gln, Cys2340Arg, and Arg2379Cys variants, there was virtually no mutant monomer secreted, with the ΔA1-band the predominant species visible, indicating that these mutations result in VWF molecules that cannot be incorporated into secreted multimers and that the rescued VWF expression in our HEK293T cells expression system represents mostly expression of wtVWF (Figure 2B, C). Together these data demonstrate that mutations in the D4 and C-domains can have a dramatic impact on VWF expression and secretion.

### 3.4 D4 and C-domain variants give rise to abnormal intracellular multimers

**Figure 3:**
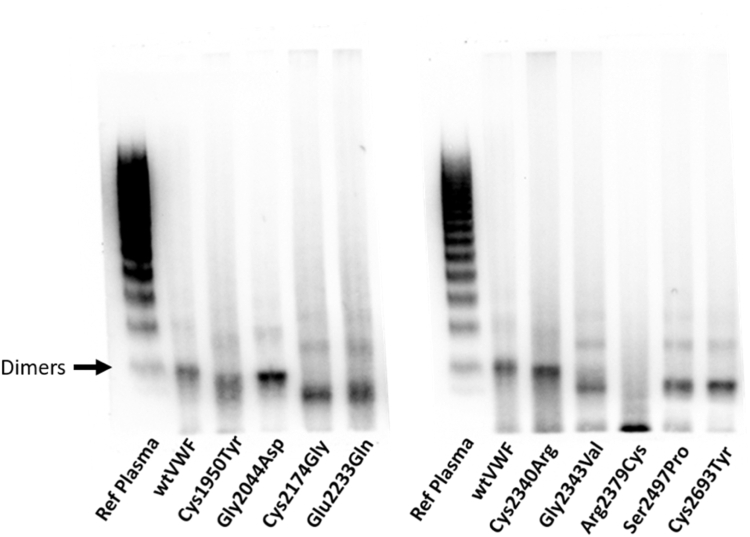
Multimeric composition of the von Willebrand disease (VWD) mutant lysates. The multimeric pattern of the variants in the cell lysate was assessed by 1.2% nonreducing agarose gel electrophoresis. Samples were diluted to 5 μg/mL of von Willebrand factor (VWF) and 10 μL loaded onto the gel reference plasma as control. The gel was subsequently visualized by western blotting with polyclonal anti-VWF horseradish peroxidase antibody. All mutants demonstrated dimers and some low molecular weight multimers except Arg2379Cys, which mainly presented monomers. Dimer bands for Cys1950Tyr, Cys2174Gly, Glu2233Gln, Gly2343Val, Ser2497Pro, and Cys2693Tyr migrated further compared with wild-type (wt) VWF and reference plasma. (Representative example of multimer gel performed in duplicate).

To further characterize the C-terminal variants, multimer gel analysis was performed on cell lysates from cells transfected with the variants in the homozygous state. In keeping with previously published reports, the cell lysates contained mainly lower-order VWF species rather than a full spectrum of multimers [44] (Figure 3). Except for the Arg2379Cys variant, all mutant proteins, like wtVWF, showed the clear presence of dimers, with 1 or 2 faint bands of higher-order multimers visible, suggesting that at least some protein was trafficked to the Golgi body for multimerization to occur (Figure 3). Strikingly, however, a number of differences were observed between the variant proteins compared with wtVWF. The Cys1950Tyr, Cys2174Gly, Glu223Gln, Gly2343Val, Ser2497Pro, and Cys2693Tyr proteins presented bands that migrated faster than the equivalent wtVWF band, and this was particularly apparent for the dimer band. Moreover, although not as distinct, the Gly2044Asp and Cys2340Arg variants also exhibited faster migrating bands. These band shifts may indicate aberrant protein folding, disulphide bond formation, or differences in glycosylation. The Arg2379Cys variant showed a virtual absence of dimers with mostly monomers present in the lysate, indicating a failure to properly form dimers.

### 3.5 VWD mutations fail to form pseudo-WPBs

**Figure 4:**
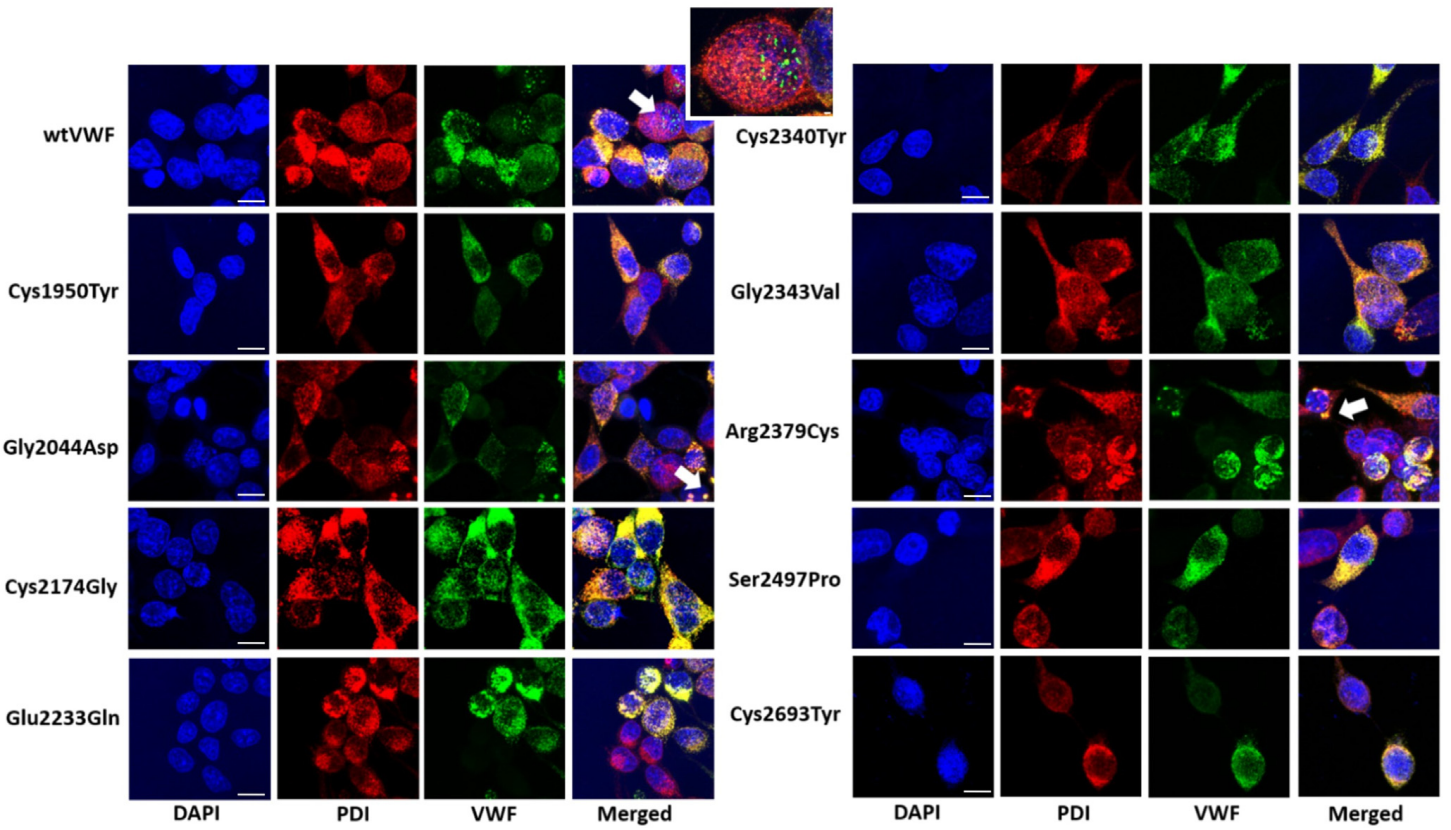
Intracellular analysis of von Willebrand disease (VWD) mutants. (A) HEK293 cells were transfected with expression vectors for wild-type von Willebrand factor (wtVWF) or the VWD mutants and subsequently fixed with 4% PFA and permeabilized with 0.1% Triton X. Cells were stained with anti-VWF fluorescein isothiocyanate (green) and antibodies against protein disulphide isomerase (PDI; red) followed by secondary Alexafluor-555–conjugated antibody. Nuclei were stained with DAPI (blue). Cells were visualized at ×63 magnification under a Zeiss LSM800 confocal microscope. All mutants demonstrated large areas of colocalization that were consistent with VWF present in the endoplasmic reticulum (ER). Pseudo-Weibel–Palade bodies (WPBs) were observed only with wtVWF (indicated by white arrow on wtVWF image). Some large punctate structures were also observed that stained positive for VWF and the ER (indicated by white arrows on the Gly2044Asp and Arg2379Cys images).

Since, in the homozygous state, all investigated mutations had either abolished or significantly reduced secretion with abnormal multimers in the cell lysate, it was next investigated whether the mutants could form pseudo-WPBs following expression in HEK293 cells. HEK293 cells were used for these experiments since the 293T counterparts fail to form pseudo-WPBs. Following transfection, cells were fixed and stained with antibodies against VWF, and the ER marker protein disulphide isomerase. HEK293 cells expressing wtVWF showed a staining pattern of VWF colocalization in the ER, and in keeping with previous data, HEK293 cells transfected with wtVWF could form distinct structures that stained positive for VWF, consistent with being pseudo-WPBs (Figure 4). Interestingly, not every cell that expressed VWF formed pseudo-WPBs with approximately 30% of cells presenting structures deemed to be pseudo-WPBs. Strikingly, none of the mutants were able to form discernible numbers of pseudo-WPBs (Figure 4), with the staining being limited to VWF localized within the ER. Another observation was that in both wtVWF and mutant-expressing cells, dense and strongly staining structures were observed that demonstrated colocalization of VWF to the ER, indicating potential areas of the ER swollen with VWF (Figure 4, white arrows). While these were present with wtVWF, they were more frequently observed with the mutants.

### 3.6 Arg2379Cys in the homozygous state is a novel type 2A mutation

**Figure 5:**
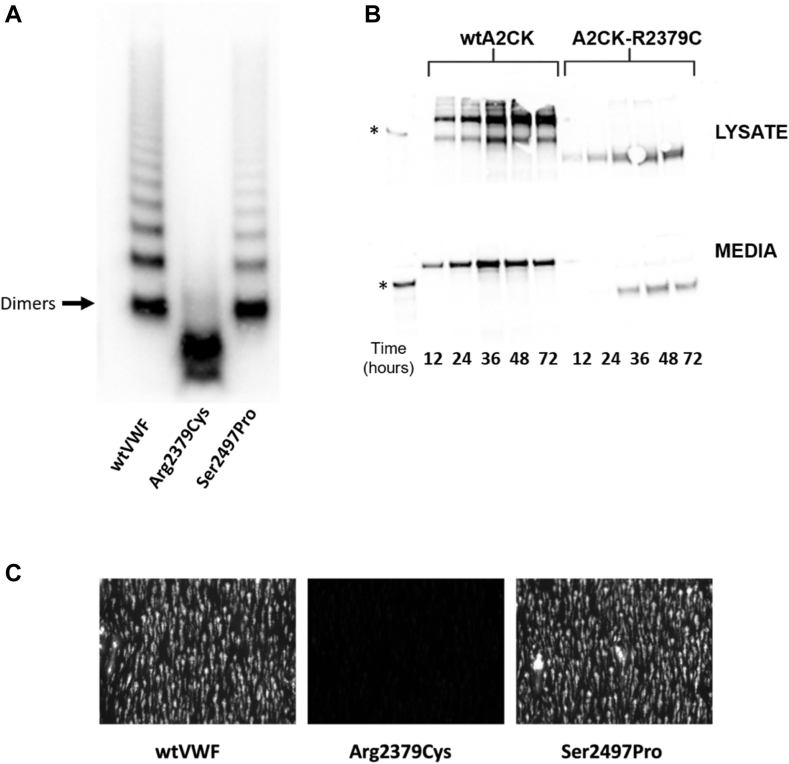
Analysis of Arg2379cys and Ser2497Pro mutants. (A) The multimeric composition of the Arg2379Cys and Ser2497Pro mutants was analyzed by nonreducing agarose gel electrophoresis. Samples were prepared using 5 μg/mL of protein, alongside wild-type von Willebrand factor (wtVWF) and reference plasma as controls. The gel was subsequently visualized by western blotting with polyclonal anti-VWF horseradish peroxidase antibody. Arg2379Cys demonstrated predominantly monomeric VWF with a feint dimer band. Ser2497Pro presented a normal multimeric profile similar to wtVWF and reference plasma. (B) HEK293T cells were transfected with expression vectors for either wtA2CK or A2CK containing the Arg2379Cys mutation. Cell lysate and media samples were harvested at different time points posttransfection and analyzed by nonreducing sodium dodecyl sulfate agarose gel electrophoresis. As a control reduced wtA2CK protein (∗) was run alongside the media and lysate samples. wtA2CK presented as both monomers and dimers in the lysate but was secreted in the dimeric form. Arg2379Cys-A2CK was only present as faster migrating monomers in the cell lysate and could only be secreted in small amounts in the monomeric form. (C) Ibidiμ–Slides V^0.1^ flow chamber slides were coated overnight with human type III collagen. Plasma-free blood was prepared by washing red blood cells and platelets, followed by labeling of platelets with DiOC_6_ for visualization. The plasma-free blood was then supplemented with 2.5 μg/mL of VWF and perfused over the collagen-coated channels at a shear rate of 1500/s. Ser2497Pro displayed a normal platelet capture profile compared with wtVWF while Arg2379Cys failed to support platelet capture. A representative image from 3 biologic replicates (*n* = 3) is depicted.

Since the Arg2379Cys and Ser2947Pro mutants could be secreted to some degree, we overexpressed these mutants and concentrated the medium to allow multimeric and functional analysis of the secreted VWF. The Ser2497Pro mutant had a normal multimeric distribution compared with wtVWF; however, consistent with the observations made with the cell lysate samples, the Arg2379Cys mutation was only present in the secreted media as mostly monomers and a faster migrating band, with a very faint dimer band visible (Figure 5A). Previously, the Arg2379Cys mutant was suggested to be a type 1 mutation [42]. However, the absence of secreted multimers suggests that in the homozygous state, Arg2379Cys is a type 2A, group I mutation. To investigate the impact of the Arg2379Cys mutation further, HEK293T cells were transfected with a VWF construct encoding the A2-CK domains and media and lysate samples were harvested at different time points posttransfection. As expected, wtVWF-A2-CK presented both monomers and dimers in the lysate that were finally secreted as dimers. In comparison, Arg2379Cys-A2-CK failed to form appreciable dimers in the cell and only monomers could be secreted, albeit at a lesser and slower extent than wtA2-CK. It was also observed that the Arg2379Cys-A2-CK bands in the cell lysate migrated further than the wtA2-CK bands, indicating either an altered structure or abnormal glycosylation (Figure 5B). Finally, to investigate the functionality of the Arg2379Cys or Ser2497Pro mutants in response to shear, flow assays were performed perfusing plasma-free blood supplemented with either wtVWF or mutant VWF and perfused over type III collagen at 1500/s. As expected, no platelet capture was observed with the Arg2379Cys mutant since it lacked functional multimers (Figure 5C). Ser2497Pro demonstrated a normal platelet capture profile similar to wtVWF, indicating that this mutation does not cause a functional defect (Figure 5C). Together, these data showed that the Arg2379Cys mutation in its homozygous state causes a type 2A phenotype by preventing dimer formation in the ER, while the Ser2497Pro mutant is unlikely to be functionally pathogenic and may be characterized as a type 1 mutation.

### 3.7 Structural impact of the VWD mutations

**Figure 6:**
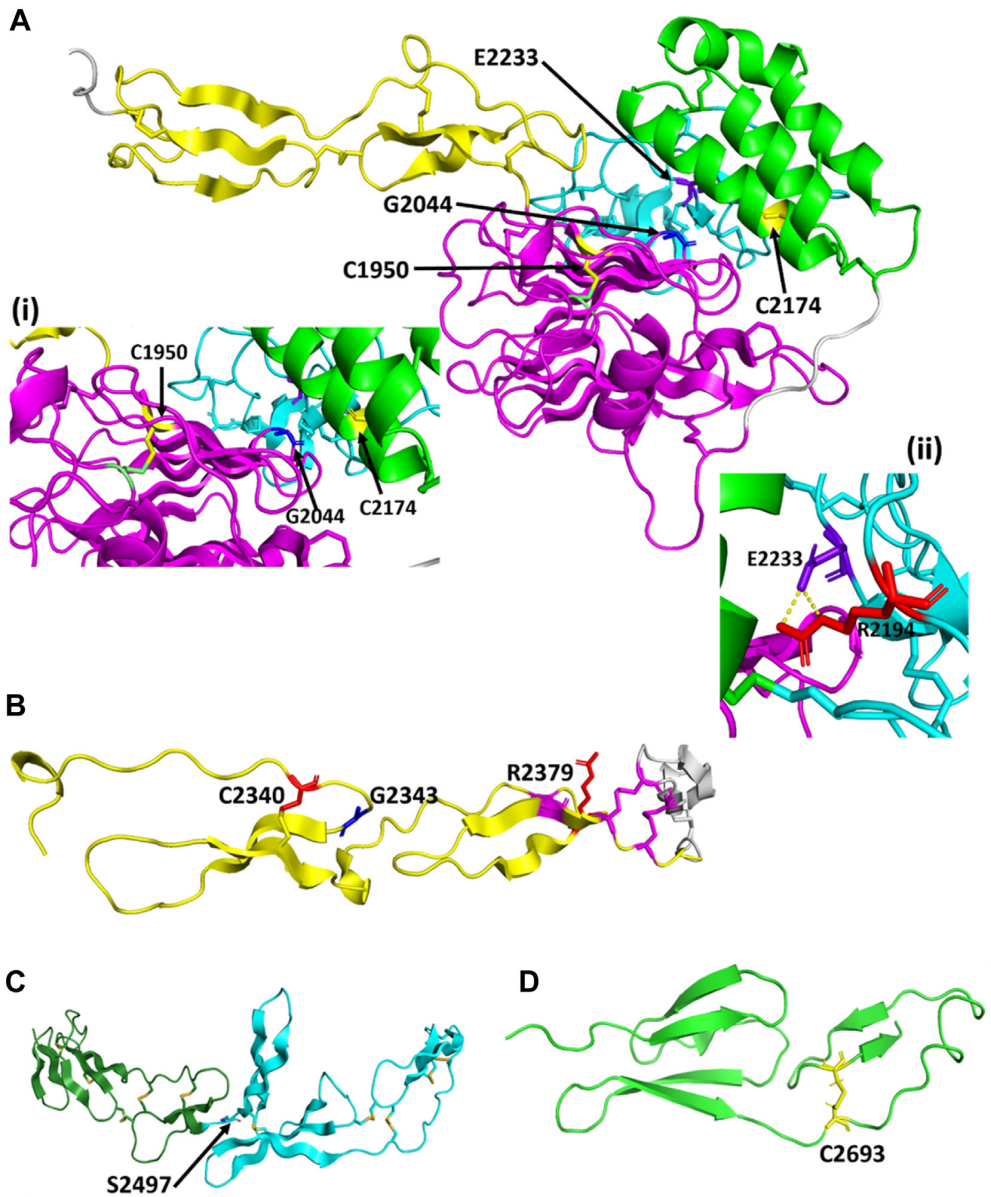
Structural analysis of von Willebrand disease (VWD) mutants. A model of the D4 assembly and the C-domains was generated using Modeller based on the porcine Alphafold template as described in materials and methods. (A) Location of mutants in the D4 assembly. The D4 assembly comprised D4N (yellow), VWD4 (magenta), C8-4 (green), and TIL-4 (cyan) is shown. Cys1950 shown in yellow occurs in the VWD4 subdomain and forms a buried disulphide bridge with Cys2085 shown in lime green. Gly2044 (shown in blue) is located at the VWD4-TIL4 interface. Cys2174 shown in yellow is located on an α-helix in the C8-4 subdomain, and Glu2233 shown in purple occurs on the TIL-4 domain. (Aii) Glu2233 forms a potential salt bridge with Arg2194, which is shown red and is located in the TIL-4 subdomain. (B) A model of the von Willebrand factor (VWF) C2 domain showing the C2 domain (yellow) and the linker region (silver). Cys2340 shown in red forms a disulphide bond with Cys2360. Gly2343 shown in blue is located at the junction of the 2 C2 subdomains. Arg2379 shown in red is located in close proximity to 3 putative disulphide bonds. (C) A model of the VWF C3C4 domains showing the C3 domain (green) and C4 domain (cyan). Ser2497 is located at the junction between the 2 domains. (D) nuclear magnetic resonance structure of the C6 domain showing Cys2693 that forms a disulphide bond with Cys2716.

To understand the impact of the mutations further, we mapped the mutations on to structural models of the D4 assembly and the C-domains. Currently, only nuclear magnetic resonance structures have been obtained for the C4 and C6 domains; thus, a model of the D4 and C-domains was generated. Based on our model, Cys1950 is located in the VWD4 part of the assembly and forms a buried disulphide bond with Cys2085 (Figure 6A). Gly2044 is also buried occurs at the interface of the VWD4 and TIL4 subdomains (Figure 6A). Mutation of both these residues is therefore likely to cause structural defects. Cys2174 is in the C8-4 domain, and our model predicts a disulphide bond with C2199 in the TIL-4 domain, with loss of this disulphide is likely to weaken the C8-4–TIL-4 interface (Figure 6A). Finally, in the D4 assembly, Glu2233 forms a potential salt bridge with Arg2194, which would be lost with the mutation (Figure 6A). In the C2 domain, Cys2340 forms a disulphide bond with Cys2360; the loss of the bond is likely to destabilize the structure (Figure 6B). The role of Gly2343 is less clear, it occurs close to the junction between the 2 subdomains of the C3 domain and could therefore affect conformation (Figure 6B). Arg2379 lies in proximity to 3 disulphide bonds, and the introduction of a new cysteine residue may therefore result in aberrant cysteine bonds forming (Figure 6B). Ser2497 occurs on the junction between C3 and C4 domains, but it is not clear how mutation of this will impact the structure (Figure 6C). Finally, Cys2693 was mapped onto the nuclear magnetic resonance structure of the C6 domain and forms a disulphide with Cys2716, and again, loss of this bond is likely to destabilize the domain (Figure 6D).

## 4 Discussion

In this study, we examined 9 mutations identified in patients with VWD, which are predicted to be causative mutations located in the D4 and C-domains of VWF. With the exception of Arg2379Cys and Ser2497Pro, all variants failed to be secreted from HEK293T cell, indicating that in the homozygous state, these mutations are highly detrimental to VWF expression. Two of the variants (Gly2044Asp and Cys2174Gly) had been previously reported in patients with type 3 VWD [40,45]. In the case of the latter, the index case was homozygous for the Cys2714Gly mutation, while the index case with the Gly2044Asp mutation was compound heterozygous with 2 other mutations Gly19Arg and Cys2491Arg [40]. Previously, we showed that Cys2491Ala cannot be secreted from HEK293T cells and, thus, a Cys2491Arg mutation would likely have the same effect [34]. Further, previous recombinant expression of the Gly19Arg did not demonstrate any effect on VWF expression [46]. The Gly2044Asp variant alone was catastrophic enough to ablate secretion and may therefore be considered a type 3 causative mutation in the homozygous state. Since the majority of patients with VWD are heterozygous, we attempted to mimic this by cotransfecting the variant VWF alongside wt. While this rescued expression to varying degrees, it was unable to restore expression to that of wtVWF alone. However, although expression can be rescued with cotransfection with wtVWF, this does not necessarily infer that the mutant VWF monomers are forming part of the secreted monomer. Cotransfections with the VWF-ΔA1 construct allowed us to distinguish mutant and normal monomers in the secreted protein. Interestingly, despite ablating secretion, Gly2044Asp was secreted in an approximately 1:1 ratio with VWF-ΔA1, thus the mutant monomers could be at least to some extent incorporated into the secreted protein. Therefore, in the reported type 3 case with Gly2044Asp, the compound heterozygous status with Cys2491Arg results in type 3 VWD. Similarly, the Glu2343Val, Ser2497Pro, and Cys2693Tyr variants could all secreted to some extent with VWF-ΔA1. For the remaining variants, only the VWF-ΔA1 band was visible, indicating that the variant monomer was unable to be rescued. It is not clear why some mutants can be rescued and others cannot, but the structural change caused by the mutations is likely to be a key factor. It is generally accepted that all cysteine residues in VWF are engaged in disulphide bond formation, except for disulphides implicated in multimer formation; this occurs within the ER and is critical for proper secretion [34,47]. Therefore, mutations affecting cysteine residues are likely to have a serious structural impact, and our structural models suggest the loss of cysteine residues is extremely likely to result in domain destabilization and misfolding and will prevent exit from the ER, even when a dimer is formed with an unaffected wt monomer. Interestingly, the Cys2693Tyr mutant was able to demonstrate rescue, indicating that the loss of this cysteine could be tolerated to some degree, possibly because this cysteine occurs toward the end of the primary amino sequence and may partake in one of the final disulphide bonds to be formed. For the remaining mutants, our structural analysis demonstrated that loss of certain residues can potentially alter subdomain interactions, especially in the D4 assembly. It should, however, be noted that with the exception of the C6 domain, these are only putative models and need to be interpreted with a degree of caution.

Multimer analysis of the cell lysates failed to reveal a full spectrum of multimers for wtVWF or the mutants; however, this is in keeping with previous observations that follow the expression of VWF in heterologous cell systems [44,48,49]. The reason for this has not been resolved. HEK293T cells secrete a full range of VWF multimers, and while the proportion of smaller-order multimeric species is generally higher than that of normal plasma, recombinant expressed VWF tends to also exhibit larger multimers than those found in plasma, presumably due to the lack of ADAMTS-13. The absence of a full multimer range in the cell lysate could represent rapid passage from the ER to the secretion pathway, and indeed, in this study, positive costaining for VWF in the Golgi body was not observed (data not shown). Apart from Gly2044Asp and Arg2379Cys, all mutants and wtVWF show the presence of dimers and 1 or 2 higher-order bands and a clear smear pattern. Nonetheless, there were some notable differences between wtVWF and the mutants. Most striking for all mutants, the dimer band migrated visibly further, indicating either defective glycosylation or altered structure. N-linked glycosylation occurs cotranslationally with the addition of the precursor glycan structure to the nascent peptide and after several trimming steps in the ER, glycosyltransferases add sugars to the glycan chains within the Golgi [50]. While altered glycosylation cannot be ruled out, it is unlikely since dimers and some higher-order species are being formed. The altered migration may therefore represent abnormal folding of the protein and, in the case of the cysteine mutations, could be due to aberrant disulphide bonds being formed.

Immunofluorescent analysis of transfected cells further confirmed the synthesis defects, with none of the mutations forming appreciable pseudo-WPBs. It is noteworthy, however, not all cells transfected with wtVWF formed WPBs, and we observed approximately 30% of cells forming WPB-like structures. Michaux et al. [44] previously reported up to approximately 60% of transfected HEK293 forming WPBs; however, other studies using the HEK293 cell model have not reported the proportion of cells expressing wtVWF. Another key observation was that the majority of the VWF staining colocalized with the ER. Although not shown, no colocalization was seen with the Golgi, although this may reflect limitations in the experimental method. Interestingly, several cells demonstrated dense, punctate areas of VWF staining that could be mistaken for WPBs; however, these colocalized with the ER marker. While these were more prominent in mutant-expressing cells, some wtVWF-expressing cells also formed similar structures. While further work is needed to precisely define the nature of these structures, they likely represent an ER that is overloaded with VWF. Interestingly, the stained structures are similar to organized smooth ER that can be formed when the ER is transformed from a network of branching tubules into stacked membrane arrays [51]. This process can occur when there is overexpression of proteins. Therefore, the overexpression of VWF in the HEK293 cell system and the enhanced ER retention observed with the variants may result in organized smooth ER formation.

Since for the Arg2379Cys and Ser2497Pro mutants some secretion was observed, these were overexpressed to obtain protein for functional analysis. Secreted Ser2497Pro formed normal multimers and behaved similar to wtVWF under flow conditions at 1500/s, indicating that no functional defects were caused by this mutation. Strikingly, the Arg2379Cys mutant failed to form multimers with only the appearance of monomers and few dimers and a band that migrated faster than the monomer. As expected, Arg2379Cys could not capture platelets under flow, consistent with the lack of multimers. When expressed as dimeric A2CK, the Arg2379Cys mutant failed to form dimers in the cell lysate and was secreted as monomers, confirming the defective dimerization. It was also observed that the Arg2379Cys monomers migrated further than the wtA2CK monomers. The introduction of a new cysteine residue is likely to have a significant impact on the protein structure. Based on our structural model of the C-domains, several cysteine residues reside in proximity to Arg2379: Cys2375, Cys2396, and Cys2398. It is probable that the new cysteine at position 2379 may potentially form an aberrant disulphide with any of these and disrupt proper protein folding. Together, these data indicate that in the homozygous state, Arg2379Cys would be a type 2A mutation that disrupts normal multimer formation. Although this variant has only yet been identified as heterozygous and is not associated with a severe bleeding phenotype, it highlights that mutations in VWF can cause different types of disease depending on their heterozygosity or homozygosity. Similarly, for the cysteine mutations characterized in this study, the heterozygous state is likely to lead to type 1 VWD with a reduction in levels of essentially normal VWF whereas, in a homozygous state or compound heterozygous state with other mutations, can lead to abolished expression. This potentially should be considered when genetically counseling patients with VWD.

Finally, although VWF clearance has not been investigated in this study, it may be postulated that heterozygous multimers may be subject to faster clearance and warrants further investigation. In summary, these data shed light on the phenotype of 9 previously described but uncharacterized mutations and highlight how mutations can behave differently depending on being homozygous or heterozygous.
